# Aromatase inhibitory activity of 1,4-naphthoquinone derivatives and QSAR study

**DOI:** 10.17179/excli2017-309

**Published:** 2017-05-16

**Authors:** Veda Prachayasittikul, Ratchanok Pingaew, Apilak Worachartcheewan, Somkid Sitthimonchai, Chanin Nantasenamat, Supaluk Prachayasittikul, Somsak Ruchirawat, Virapong Prachayasittikul

**Affiliations:** 1 Department of Clinical Microbiology and Applied Technology, Faculty of Medical Technology, Mahidol University, Bangkok 10700, Thailand; 2 Center of Data Mining and Biomedical Informatics, Faculty of Medical Technology, Mahidol University, Bangkok 10700, Thailand; 3 Department of Chemistry, Faculty of Science, Srinakharinwirot University, Bangkok 10110, Thailand; 4 Department of Community Medical Technology, Faculty of Medical Technology, Mahidol University, Bangkok 10700, Thailand; 5 Department of Clinical Chemistry, Faculty of Medical Technology, Mahidol University, Bangkok 10700, Thailand; 6 Laboratory of Chemical Carcinogenesis, Chulabhorn Research Institute, Bangkok 10210, Thailand; 7 Laboratory of Medicinal Chemistry, Chulabhorn Research Institute and Program in Chemical Biology, Chulabhorn Graduate Institute, Bangkok 10210, Thailand; 8 Center of Excellence on Environmental Health and Toxicology, Commission on Higher Education (CHE), Ministry of Education, Thailand

**Keywords:** 1,4-naphthoquinones, aromatase inhibitory activity, structural modification, computer-aided drug design, anticancer agents

## Abstract

A series of 2-amino(chloro)-3-chloro-1,4-naphthoquinone derivatives (**1**-**11**) were investigated for their aromatase inhibitory activities. 1,4-Naphthoquinones **1** and **4** were found to be the most potent compounds affording IC_50 _values 5.2 times lower than the reference drug, ketoconazole. A quantitative structure-activity relationship (QSAR) model provided good predictive performance (*R^2^*_CV _= 0.9783 and RMSE_CV_ = 0.0748) and indicated mass (Mor04m and H8m), electronegativity (Mor08e), *van der* Waals volume (G1v) and structural information content index (SIC2) descriptors as key descriptors governing the activity. To investigate the effects of structural modifications on aromatase inhibitory activity, the model was employed to predict the activities of an additional set of 39 structurally modified compounds constructed *in silico*. The prediction suggested that the 2,3-disubstitution of 1,4-naphthoquinone ring with halogen atoms (i.e., Br, I and F) is the most effective modification for potent activity (**1a**, **1b** and **1c**). Importantly, compound **1b **was predicted to be more potent than its parent compound **1** (11.90-fold) and the reference drug, letrozole (1.03-fold). The study suggests the 1,4-naphthoquinone derivatives as promising compounds to be further developed as a novel class of aromatase inhibitors.

## Introduction

The direct inhibition of estrogen synthesis targeting aromatase inhibition is considered to be an effective strategy towards breast cancer treatment (Altundag and Ibrahim, 2006[1]; Brueggemeier et al., 2005[5]; Favia et al., 2013[12]). The majority of available non-steroidal aromatase inhibitors exhibit their competitive inhibitory activity through the coordination of the nitrogen (N) atom presented in the molecule with the iron heme atom of the enzyme (Recanatini et al., 2002[36]). Currently, aza-based aromatase inhibitors, i.e., letrozole and anastrozole, have been approved by the FDA as standard drugs for breast cancer treatment. In addition, natural products, i.e., flavonoids (Kao et al., 1998[18]; Karjalainen et al., 2000[19]; Le Bail et al., 2001[23]) and sesquiterpene lactones (Blanco et al., 1997[3], 2001[4]), whose carbonyl oxygen (O) atoms have been noted to play a role in heme coordination (Kao et al., 1998[18]). 

1,4-Naphthoquinone scaffold is well known for its chelating ability and electrochemical properties (Hillard et al., 2008[15]; Martinelli et al., 1999[28]). It contains two ketone groups as crucial moieties, which are responsible for many biological activities because of their abilities to accept electrons (Benites et al., 2010[2]; O'Brien, 1991[32]). Various biological activities of 1,4-naphthoquinone derivatives have been reported, i.e., anticancer (Kishore et al., 2014[20]; Mallavadhani et al., 2014[26]; Nasiri et al., 2013[31]; Prachayasittikul et al., 2014[35]; Sreelatha et al., 2014[42]), radical scavenging (Kumar et al., 2013[21]; Lebedev et al., 2008[24]; Song et al., 2000[40]), antimicrobial (Ryu and Kim, 1992[37]; Sreelatha et al., 2014[42]; Tandon et al., 2009[45]), antiviral (Crosby et al., 2011[9]; Da Costa et al., 2013[10]; Ilina et al., 2002[16]), antifungal (Castro et al., 2013[6]; Pawar et al., 2014[33]; Tandon et al., 2009[45]), antitrypanocidal (Salmon-Chemin et al., 2001[38]) and antiplatelet (Jin et al., 2004[17]; Kuo et al., 2011[22]; Lien et al., 2002[25]; Yuk et al., 2000[48]) activities. It was found that structural modification can effectively modulate the electrochemical behavior of 1,4-naphthoquinone compounds, thereby affecting their biological activities (Hillard et al., 2008[15]). The introduction of amino and/or chloro moieties into the core structure of 1,4-naphthoquinone was documented to improve the redox potential of the compounds (Benites et al., 2010[2]; Salunke-Gawali et al., 2014[39]). 

Computational approaches have been recognized as fundamental tools for facilitating drug design and development (Mandal et al., 2009[27]; van de Waterbeemd and Gifford, 2003[46]). Quantitative structure-activity relationship (QSAR) has been extensively employed to facilitate lead optimization (Cherkasov et al., 2014[7]; Cramer, 2012[8]). QSAR correlates the chemical structure of compounds with their biological activities (Nantasenamat et al., 2009[29], 2010[30]; Prachayasittikul et al., 2014[35]), which provides information about influential chemical features, crucial moieties and pharmacokinetic properties (Hansch et al., 2004[14]; Prachayasittikul et al., 2014[35]). 

**Figure 1:**
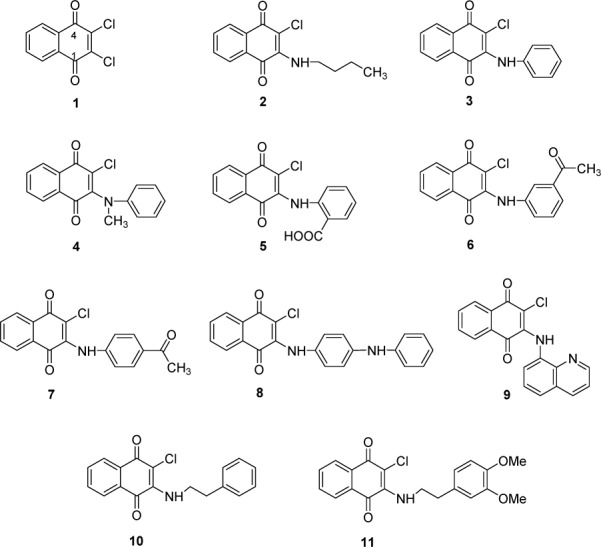
Chemical structures of 1,4-naphthoquinone derivatives (1-11)

**Figure 2:**
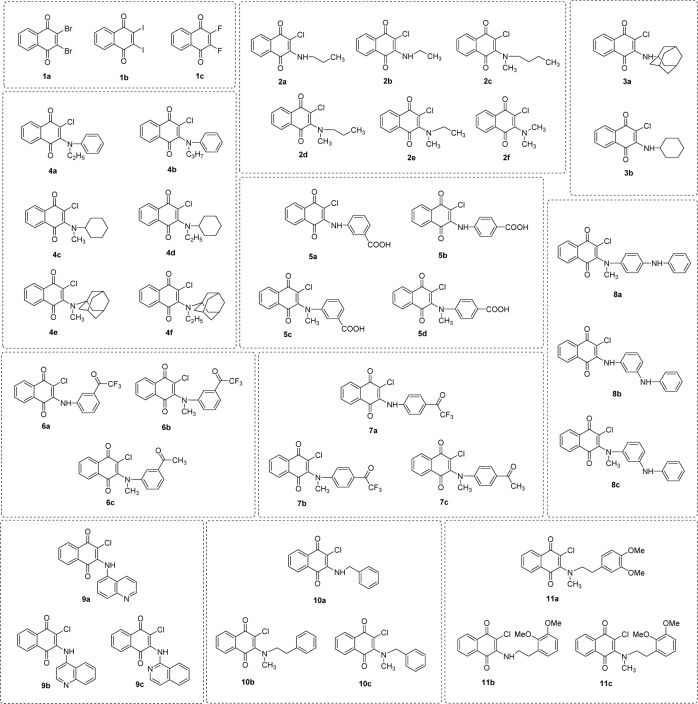
Chemical structures of structurally modified compounds (series 1-11)

To date, 1,4-naphthoquinone derivatives have not been reported as aromatase inhibitors. Based on the heme coordination mechanism, 2-amino-1,4-naphthoquinone compounds are considered to be a potential novel class of aromatase inhibitors because of the presence of heme coordinating atoms, i.e., amino N and carbonyl O atoms in their molecules. Recently, a series of 2-amino-3-chloro-1,4-naphthoquinones was reported to possess anticancer activity by our research group (Prachayasittikul et al., 2014[35]). Herein, a set of eleven 2-amino(chloro)-3-chloro-1,4-naphthoquinone compounds (**1**-**11**, Figure 1(Fig. 1)) were investigated for their aromatase inhibitory activities, and the experimental activities of the compounds (**1**-**11**) were used for QSAR analysis. To elucidate the effects of structural modification on the core structure of 2,3-disubstituted-1,4-naphthoquinone, an additional set of 39 structurally modified compounds derived from compound series **1**-**11** (Figure 2(Fig. 2)) were constructed *in silico*, and their aromatase inhibitory activities were predicted using the constructed QSAR model. 

## Materials and Methods

### Compounds and reagents 

A series of 1,4-naphthoquinone derivatives (**1**-**11**) were synthesized as by a previously described method (Prachayasittikul et al., 2014[35]). A recombinant human aromatase (CYP19) and *O-*benzyl fluorescein benzyl ester (DBF) were supplied with the BD Gentest^TM ^kit from BD Biosciences-Discovery Labware (Woburn, USA). Dimethyl sulfoxide (DMSO) was obtained from EMD Millipore (Billerica, MA, USA). 

### Aromatase inhibition assay

The aromatase inhibitory activity of compounds **1**-**11** was investigated by the method previously described by Stresser et al. (Stresser et al., 2000[43]), with minor modifications (Prachayasittikul et al., 2014[34]). The assay was performed with a Gentest kit using enzyme CYP19 and DBF as a fluorometric substrate. DBF was dealkylated by aromatase and then hydrolyzed, yielding the fluorescein product. Briefly, 100 µL of cofactor containing 78.4 µL of 50 mM phosphate buffer (pH 7.4), 20 µL of 20x NADPH-generating system (26 mM NADP^+^, 66 mM glucose-6-phosphate and 66 mM MgCl_2_), and 1.6 µL of 100 U/mL glucose-6-phosphate dehydrogenase was pipetted into a 96-well plate and preincubated in a water bath (37 °C) for 10 min. The reaction was initiated by adding 100 µL of an enzyme/substrate mixture containing 77.3 µL of 50 mM phosphate buffer (pH 7.4), 12.5 µL of 16 pmol/mL CYP19, 0.2 µL of 0.2 mM DBF and 10 µL of tested compound or 10 % DMSO as a negative control or ketoconazole/letrozole as a positive control. After the incubation at 37 °C for 30 min, the reaction was terminated by adding 50 µL of 2.2 N NaOH. The fluorescence signal was recorded using an excitation wavelength of 490 nm and an emission wavelength of 530 nm with a cutoff of 515 nm. The percentage of inhibition (% inhibition) was calculated using Equation 1. Compounds with greater than 50 % inhibition were determined to be active compounds and were further diluted and assayed in triplicate. IC_50_ values were determined by plotting concentrations versus % inhibition.


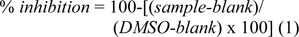


### QSAR analysis

**Figure 3:**
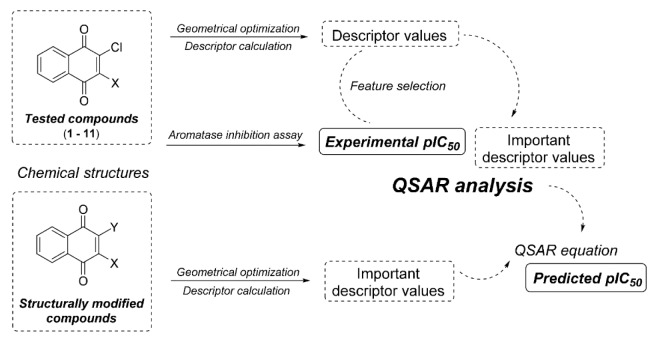
Workflow of the QSAR study

Conceptually, a QSAR model was constructed using the data obtained from experimentally tested compounds (**1**-**11**). The constructed model was subsequently used to predict the activity of the additional set of structurally modified compounds (series **1**-**11**) that were generated *in silico. *The conceptual workflow of the QSAR study is shown in Figure 3(Fig. 3).

#### Data set

Chemical structures of the tested compounds (**1**-**11**) along with their experimental IC_50 _values were used to construct the QSAR model. The IC_50 _values were converted to pIC_50_ values by taking the negative logarithm to base 10 (-log IC_50_) to obtain a normal distribution of data points. Experimentally inactive compounds were excluded from the data set. 

#### Geometry optimization and descriptor calculation

The chemical structures of 11 tested compounds (**1**-**11**) and 39 structurally modified compounds (series **1**-**11**) were drawn using the GaussView software (Dennington et al., 2003[11]). Optimization was performed to obtain the low-energy conformers for further calculation of molecular descriptor values. The compounds were geometrically optimized using Gaussian 09 (Frisch et al., 2009[13]) at the semi-empirical Austin Model 1 (AM1) level, followed by density functional theory (DFT) calculation using Becke's three-parameter hybrid method with the Lee-Yang-Parr correlation functional (B3LYP) together with the 6-31g(d) level. A set of 13 quantum chemical descriptors, i.e., mean absolute atomic charge (*Q*_m_), total energy (*E*_total_), total dipole moment (*μ*), highest occupied molecular orbital energy (*E*_HOMO_), lowest unoccupied molecular orbital energy (*E*_LUMO_), energy difference of HOMO and LUMO (HOMO-LUMO_Gap_), electron affinity (EA), ionization potential (IP), Mulliken electronegativity (*χ*), hardness (*η*), softness (*S*), electrophilic index (*ω*_i_) and electrophilicity (*ω*), was extracted using a script developed in-house. The optimized structures were further calculated to obtain an additional set of 3,224 molecular descriptors using Dragon software version 5.5 (Talete, 2007[44]). The molecular descriptors obtained from the Dragon software included 22 classes, i.e., Constitutional descriptors, Topological descriptors, Walk and path counts, Connectivity indices, Information indices, 2D autocorrelation, Edge adjacency indices, Burden eigenvalues, Topological charge indices, Eigenvalue-based indices, Randic molecular profiles, Geometrical descriptors, RDF descriptors, 3D-MoRSE descriptors, WHIM descriptors, GETAWAY descriptors, Functional group counts, Atom-centered fragments, Charge descriptors, Molecular properties, 2D binary fingerprints and 2D frequency fingerprints.

#### Feature selection

Correlation-based feature selection was employed to initially select important descriptors from a large set of descriptors obtained from the calculation. The pair-correlation of each descriptor value and bioactivity (pIC_50_) was calculated using a Pearson's correlation coefficient (r) of 0.6 as a cut-off value. Descriptors with |r| < 0.6 were considered weakly correlated descriptors and were excluded from the study, whereas those with |r| ≥ 0.6 were selected for further selection process using stepwise multiple linear regression (MLR) as implemented in SPSS statistics 18.0 (SPSS Inc., USA[41]). Finally, a set of important descriptors was obtained for multivariate analysis. 

#### Multivariate analysis 

Multivariate analysis was performed by Waikato Environment for Knowledge Analysis (WEKA) version 3.4.5 (Witten et al., 2011[47]) using a multiple linear regression (MLR) algorithm. Selected descriptor values and pIC_50 _values were assigned as independent variables (*X*) and dependent variables (*Y*), respectively. The MLR model was constructed according to Equation 2: 


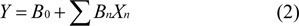


where *Y* represents the pIC_50_ values of the compounds, *B_0_* is the intercept and *B_n_* are the regression coefficients of descriptors *X_n_*.

#### Data sampling

The data set was divided into a training set and a testing set by means of leave-one-out cross validation (LOO-CV). Conceptually, one sample was removed from the entire data set (*N*) and was used as the testing set, whereas the remaining samples (*N*-1) were used as the training set. The same sampling process was continued until every sample in the data set was used as the testing set to predict the variable *Y *(activity). 

#### Evaluating the performance of QSAR model

Two statistical parameters were used to assess the predictive performance of the constructed QSAR model. The squared correlation coefficient (*R^2^*) represented the predictive performance, and the root mean square error (RMSE) represented the predictive error of the model. 

#### Prediction of structurally modified compounds (series 1-11)

All structurally modified compounds were drawn, optimized and calculated to obtain a set of important descriptor values as describe above. The QSAR equation obtained from the QSAR analysis of tested compounds (**1**-**11**) was used to calculate the predicted activity of the modified compound series. The descriptor values of modified compounds were replaced in the equation by independent variables (*X)* to predict their aromatase inhibitory activity (pIC_50_). 

## Results and Discussion

### Aromatase inhibitory activity 

The aromatase inhibitory activity of the quinone compounds (**1**-**11**) are summarized in Table 1(Tab. 1). The compounds were categorized according to their aromatase inhibitory activities (IC_50_) as highly active (IC_50_ < 1 µM), moderately active (1 µM < IC_50 _< 10 µM), weakly active (IC_50_ > 10 *µ*M) and inactive (% inhibition ≤ 50 % at 12.5 µM) (Prachayasittikul et al., 2014[35]). The results indicate that compounds **1** and **4** are the most potent compounds, affording equivalent IC_50_ values of 0.5 ± 0.3 and 0.5 ± 0.4 µM, respectively. Both compounds (**1** and **4**) exhibited more potent aromatase inhibitory activity than did the reference drug, ketoconazole, as indicated by their IC_50 _values, which were 5.2-fold lower than the value obtained for ketoconazole (IC_50 _= 2.6 ± 0.7 µM). Compounds **3** and **11 **were found to be inactive, and the rest of the tested compounds exhibited moderately active (**2**, **6**, **7**, **8**, **9** and **10**) to weak (**5**) activities. The order of aromatase inhibitory activity was as follows: **1** ≈ **4 **> **8** > **6 **> **10** > **9** > **7** > **2** > **5** >> **3** and **11**. A detailed discussion regarding the structure-activity relationships of the tested compounds is provided in Supplementary information.

The cytotoxicity of compounds **1**-**11 **against the normal MRC-5 cell line (Table 1(Tab. 1)) was previously documented (Prachayasittikul et al., 2014[35]). Moderately active compounds **2** and **10** and inactive compound **11** were shown to be non-cytotoxic. It should be noted that both of the highly active aromatase inhibitors (**1** and **4**) exhibited a high safety index with selectivity index values of 45.98 and 17.20, respectively. However, compound **4 **displayed higher cytotoxicity than compound **1**. 

### QSAR analysis of naphthoquinones 1 - 11

A set of five informative descriptors (i.e., Mor04m, Mor08e, H8m, G1v and SIC2) was obtained using correlation-based feature selection. Definitions of the selected descriptors and the descriptor values of the investigated compounds are presented in Table 2(Tab. 2) and Supplementary Table S1, respectively. The QSAR model (Equation 3) was successfully constructed using a multiple linear regression (MLR) algorithm. 


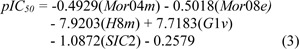


**Figure 4:**
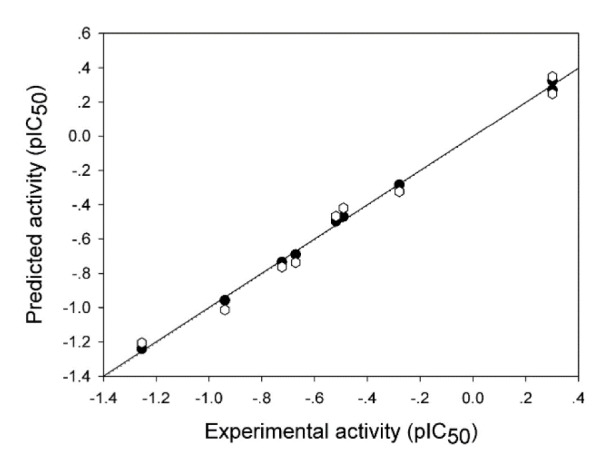
Plot of experimental versus predicted pIC_50 _values of aromatase inhibitory activity of the tested compounds generated by the QSAR model (training set: compounds are represented by filled circles, and the regression line is shown as a solid line; leave-one-out validated testing set: compounds are represented by open hexes, and the regression line is shown as a dotted line).

The model provided suitable predictive performance, affording *R^2^*_Tr _= 0.9984 and RMSE_Tr _= 0.0192 for the training set and* R^2^*_CV_ = 0.9783 and RMSE_CV_ = 0.0748 for the testing set. The experimental and predicted activities (pIC_50 _values) of the compounds (**1 **- **11**) in the data set are summarized in Supplementary Table S2 and Figure 4(Fig. 4). 

The QSAR analysis (Equation 3) revealed that the mass (Mor04m and H8m), electronegativity (Mor08e), *van der* Waals volume (G1v) and structural information content index (SIC2) descriptors are influential descriptors governing the aromatase inhibitory activity of the compounds. The mass-weighted descriptor H8m was found to be the most influential descriptor, as indicated by the highest regression coefficient of -7.9203. The negative regression coefficient indicated that the low value of H8m is required for potent activity. Similarly, low values are required for other important descriptors with negative regression coefficients, i.e., Mor04m, Mor08e, and SIC2, whereas the high value of the descriptor with a positive regression coefficient, i.e., G1v, is required for suitable activity. A comprehensive analysis of SAR was performed to elucidate the effects of substituents on important descriptor values and aromatase inhibitory activity, the results of which are summarized in Supplementary Table S3. 

Compound **1** (2,3-dichloro-1,4-naphthoquinone) was used as a prototype or a parent compound for comparison with its derivatives as 2-amino-3-chloro-1,4-naphthoquinones (**2**-**11**). It was found that compound **4 **was the only one that exhibited experimental activity comparable to that of compound** 1** (highly active), whereas the rest of the compounds displayed lower potent activity (moderately to weakly active) (Table 1(Tab. 1)). The experimental results indicate that the replacement of the 2-chloro group of **1** with amino phenyl ring leads to compound **3 **with a loss of activity (Table S3, Panel 2). The deleterious effects may be due to an alteration of the mass, electronegativity and *van der* Waals volume of the compound, as indicated by the Mor04m, Mor08e and G1v values (Table S1 and Table S3, Panel 2). A remarkable reduction in G1v but increases in Mor04m and Mor08e were noted for the inactive compound **3** compared to the most potent compound **1**, which showed lower Mor04m (1.226) and Mor08e (-0.928) values but higher G1v (0.193) values (Table S1). Such descriptor values are well correlated with the results of the QSAR model, as previously mentioned. However, improved activities were observed when various types of substituents were placed on the 2-amino group (Table S3, Panel 3) or on the phenyl ring of the 2-amino group (Table S3, Panels 4 - 7). In particular, the highly potent activity of the compound** 4 **is governed by the additional substitution of the CH_3_ group on the 2-amino group of inactive compound **3**, which causes the opposite effect on related descriptors (i.e., Mor04m, Mor08e and G1v) such that it enhances the activity (Table S3, Panel 3). Compound **4 **showed a higher G1v value (0.190) but lower Mor04m (1.104) and Mor08e (-0.728) values compared with those of compound **3**, which showed a lower G1v (0.619) value but higher Mor04m (1.672) and Mor08e (-0.494) values. 

In comparing *N-*phenyl compounds (**5**-**8**) with compound **4 **(Table S1), it was found that compound **8 **showed higher values of Mor04m (1.499), Mor08e (-0.478) and H8m (0.021) but a lower G1v value (0.169) than the most potent compound **4 **(Mor04m = 1.104, Mor08e = -0.728, H8m = 0.002 and G1v = 0.190). Similar effects were noted for compounds** 5**-**7**, which showed higher Mor04m, Mor08e and H8m values but lower G1v values than compound **4**. Such high values of mass (Mor04m and H8m) and electronegativity (Mor08e) descriptors could be due to the effects of substituents (R= NHPh, COCH_3_, and CO_2_H) on the Ph ring of secondary amines (**5**-**8**), and lower values of *van der* Waals volume were observed when comparing with the tertiary amine **4**. Among the compounds **5**-**8**, the one with an electron-donating group at the *para*-position (**8**, R = NHPh) had a lower Mor08e value (-0.478) compared to that with an electron-withdrawing group at the *para*-position (**7**, R = COCH_3_, Mor08e = -0.280). Compound **6,** with R (COCH_3_) at the *meta*-position, showed a lower Mor08e value (-0.642) compared with its *para*-isomer (**7**). For the *ortho*-CO_2_H group (R) of compound **5**, a higher Mor08e value (-0.182) was observed. The results suggest that the electronic effects of R groups provided a Mor08e value that is well correlated with the activity, in which a lower Mor08e value yielded a higher activity for the compounds, as noted for compounds **8** > **6** > **7** > **5** (Table S1).

Likewise, the substitution of the 2-chloro group on the naphthoquinone ring by an amino alkyl chain can reduce the activity of the compound, as observed for compound **2** (Table S3, Panel 1). The conversion of the *N*-alkyl chain of compound **2 **to an *N*-alkylphenyl group afforded compound **10** with an increased G1v value but decreased Mor04m and Mor08e values that improved the activity of the compound. On the other hand, the introduction of diOCH_3_ into the terminal phenyl ring of compound **10** yielded inactive compound **11** with a decreased G1v value. Interestingly, compound **10** (IC_50_ = 3.3 µM) exhibited relatively high and comparable activity to that of compound **6** (IC_50_ = 3.1 µM). This finding could be attributed to the flexible ethyl phenyl group on the 2-amino position of compound **10** (low Mor08e or electron-donating effect but high G1v values) that causes the molecule to exist in a more favorable form when interacting with the target site. In contrast, the diOCH_3_ groups of compound **11** might produce a bulky molecule that is unfavorable for exerting the activity. *N-*anilinyl analog **9** (Table S3, Panel 8) showed a lower Mor08e value (-1.001) but a higher G1v value (0.179) than did *N*-phenyl analog** 3 **(Mor08e = -0.494, G1v = 0.169), thereby giving rise to more potent activity for the former. 

### Prediction of structurally modified compounds (series 1-11)

The predicted activities of all modified compounds are presented in Table S4. Similarly to the tested compounds, the modified compounds were categorized according to their predicted activity (Prachayasittikul et al., 2014[35]). The majority of the modified compounds were predicted as moderately active aromatase inhibitors. Some of the modified compounds (Table S4) exhibited more potent activity than the reference drug ketoconazole but less than letrozole (i.e., **1a**, **1c**, **2f**, **3a**, **4a**, **4b**, **4d**, **4e**, **4f**, **5d**, **6c**, **7b**, **7c**, **8a**, **8b** and **8c**). 

The predictions showed that structural modifications can either improve or deteriorate the aromatase inhibitory activity of the compounds. 2,3-Disubstitution of the 1,4-naphthoquinone ring with halogen atoms (i.e., Br, I and F) was found to be the most effective strategy, as indicated by the highly potent activity of all modified compounds in series** 1 **(Table S4). Importantly, compound **1b **exhibited the most potent activity among the tested and modified compounds, affording pIC_50_ values 11.90-fold and 1.03-fold greater than those of the parent compound **1 **(experimental pIC_50_ 0.301, Table S2) and the reference drug letrozole (experimental pIC_50_ 3.482, Table S4), respectively. Greatly improved activity was also observed in other compounds in the same series, such as compound **1c** (predicted pIC_50_ 0.614, Table S4). This compound was ranked as the second most potent aromatase inhibitor, in which the activity was improved 2.04-fold compared to that of the parent compound** 1**. Likewise, compound **1a **(predicted pIC_50_ 0.550, Table S4) was the third most potent compound, with a 1.83-fold improvement in activity compared to that of parent compound **1**. The effects of structural modifications were elucidated by analyzing the relationship between descriptor values and activity, as summarized in Supplementary Table S5. It could be deduced that the markedly improved activities of the modified compounds in series **1** may be governed by the substituted halogen atoms, which reduce the mass-weighted descriptor (Mor04m) values of the compounds. In particular, the lowest Mor04m value (-5.734) was observed for the most potent compound **1b**, whereas the parent compound **1** exhibited the highest Mor04m value (1.226), followed by compounds **1a** (0.479) and **1c** (0.465). It should be noted that the modified compounds (**1a**, **1b**, **1c**) and the parent compound **1 **showed the same values for the descriptors H8m, SIC2, and G1v (i.e., 0, 0.707 and 0.193, respectively). However, these compounds showed different Mor08e values: **1b** = -0.583, **1a** = -0.644, **1c** = -0.757 and **1** = -0.928 (Table S1).

Mono-substitution of the 2-chloro group in the core structure (compound **1**) by amino-based moieties produced 2-amino-3-chloro compounds with diverse effects. Compared with the activity of compound **1**, reduced activities were observed in all modified compounds in series **2**-**11**, except for compound **4a **(predicted pIC_50_ 0.326, Table S4), which exhibited more potent activity, as indicate by a pIC_50_ value that was 1.08-fold greater than that of the parent compound **1** but 10.68-fold less potent than the reference drug, letrozole. 

Results obtained from the modified compounds in series **4** indicated that the length of the substituted alkyl chain (R^1^) and type of substituted ring (R^2^) on the amino group at the C-2 position of the 1,4-naphthoquinone core affected the aromatase inhibitory activity of the compounds *via *the alterations of mass (Mor04m and H8m), electronegativity (Mor08e) and *van der* Waals volume (G1v). Substitution with the phenyl group (R^2^) of compounds **4a** (R^1^ = C_2_H_5_) and **4b** (R^1^ = C_3_H_7_) and with the 1-adamantyl group (R^2^) of compounds **4e** (R^1^ = CH_3_) and **4f** (R^1^ = C_2_H_5_) on the amino moiety led to highly potent compounds, whereas the compounds substituted with a cyclohexyl ring (R^2^), i.e., **4c **(R^1^ = CH_3_) and **4d** (R^1^ = C_2_H_5_), exhibited less potent activity. Apparently, substitution with a 2C (C_2_H_5_) alkyl chain (R^1^) in combination with a phenyl ring (R^2^) yielded a tertiary amine (**4a**), which was considered to be the most appropriate modification of 1,4-naphthoquinones with 2-substituted amino moieties. Among the modified compounds in series** 4**, compound **4a** showed the lowest H8m value (0.002) but a relatively high G1v value (0.162). 

The effects of *ortho*-/*meta*-/*para*-anilinyl (C_6_H_6_N) substituted onto the amino phenyl ring along with the presence of an additional methyl (CH_3_) substituent on the 2-amino group of the 1,4-naphthoquinone core were investigated in modified compound series **8**. The *para*-aniline compound **8** (pIC_50_ -0.279) exerted more potent activity than did the *meta*-compound **8b** (pIC_50_ -0.390), which could be due to the alteration of the mass descriptor, H8m (**8** = 0.021, **8b** = 0.002, Table S1). Furthermore, remarkable enhancement effects were observed when the methyl group was introduced into the 2-amino position of both the *para*- (**8a**, pIC_50_ 0.259) and *meta*- (**8c, **pIC_50_ 0.123) aniline compounds, among which **8a** was shown to be the most potent compound. The markedly increased activity of *N*-methyl-substituted compounds **8a** and **8c** may be governed by the mass descriptor, as indicated by the 0.49-fold and 0.33-fold decreases in their Mor04m values compared with those of non-substituted *N*-methyl compounds **8** and **8b**, respectively. Likewise, the effects of *ortho*-/*meta*-/*para*-carboxyl (COOH) and acetyl (COCH_3_) substitutions on the amino phenyl ring were investigated in modified compound series **5**, **6**, and **7 **(detailed discussion is provided in Supplementary information).

Similarly, the activity of modified compound series **2**, **3**, **9**, **10** and **11** were affected by other factors, i.e., the length of the alkyl chain, methyl substitution at the 2-amino group, diOMe substitution on the terminal phenyl ring, and type of substituted ring (detailed discussion is provided in Supplementary information).

Finally, the comprehensive SAR analysis of both tested and modified compounds revealed that certain functional groups substituted in a particular position and/or in a distinct combination are essential for improving the aromatase inhibitory activity of the compounds by altering important descriptor values governing the activity (Supplementary Table S4).

## Conclusion

A series of 2,3-disubstituted-1,4-naphthoquinone derivatives (**1**-**11**) were investigated for their aromatase inhibitory activities. Compounds **1** (2,3-dichloro) and **4 **(2-amino-3-chloro) exhibited the most potent activity, affording IC_50 _values 5.2-fold lower than that of the reference drug, ketoconazole. The QSAR study revealed that mass (Mor04m and H8m), electronegativity (Mor08e), *van der* Waals volume (G1v) and structural information content index (SIC2) descriptors are influential descriptors governing aromatase inhibitory activity. The 2,3-dihalogen derivatives (**1a**, **1b** and **1c**) were predicted to be the most potent modified series, affording predicted pIC_50_ values in range of 0.550 - 3.582. The prediction suggested that considerably improved activity can be achieved when the 2,3-position of the 1,4-naphthoquinone rings are substituted by halogen atoms with high lipophilicity and electronegativity (i.e., Br, I and F). Notably, the 2,3-diiodo compound **1b** exhibited the most potent predicted activity affording the pIC_50_ value 11.90-fold and 1.03-fold greater than those of its parent compound **1** and the reference drug (letrozole), respectively. Finally, this study provides pertinent knowledge regarding drug design and development and suggests that 1,4-naphthoquinone-based compounds can be further developed as a novel class of aromatase inhibitors. 

## Notes

Supaluk Prachayasittikul and Virapong Prachayasittikul (E-mail: virapong.pra@mahidol.ac.th) contributed equally as corresponding authors.

## Supplementary information

Supplementary information is available on the EXCLI Journal website.

## Acknowledgements

This project is supported by the Office of the Higher Education Commission, Mahidol University under the National Research Universities Initiative and Annual Government Grant under Mahidol University (2556-2558 B.E.). We gratefully acknowledge the Chulabhorn Research Institute for bioactivity experiments.

## Conflict of interests

The authors declare that they have no conflicts of interest.

## Supplementary Material

Supplementary information

## Figures and Tables

**Table 1:**
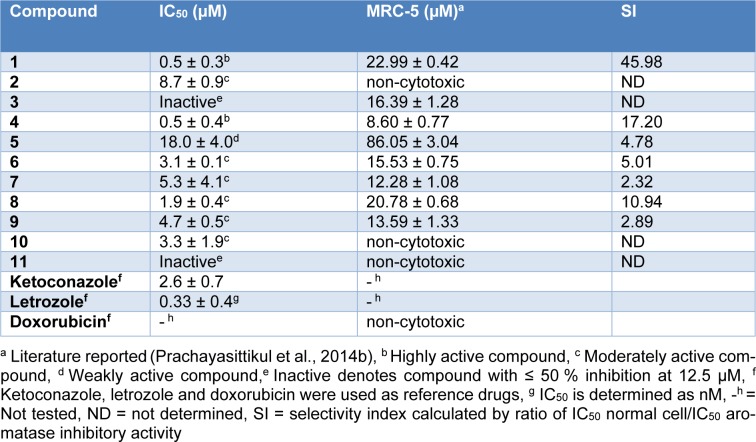
Aromatase inhibitory activity (IC_50_) of 1,4-naphthoquinone derivatives (1-11)

**Table 2:**
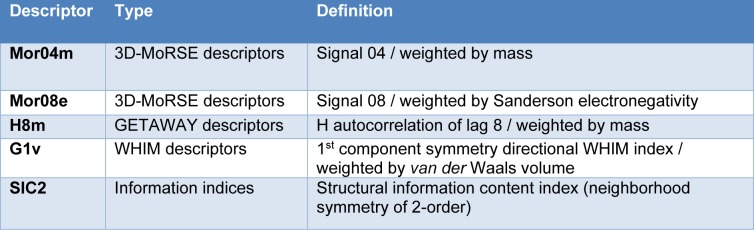
Definition of descriptors used for development of QSAR models
