# Supplementary material for: Aromatase inhibitory activity of 1,4-naphthoquinone derivatives and QSAR study
Source: EXCLI J. 2017 May 16;16:714–26. doi: 10.17179/excli2017-309 (PMC5547393; doi:10.17179/excli2017-309)
Supplement: Supplementary information [file EXCLI-16-714-s-001.pdf]

EXCLI Journal 2017;14:714-726 – ISSN 1611-2156
Received: March 28, 2017, accepted: May 02, 2017, published: May 16, 2017

Supplementary information to

AROMATASE INHIBITORY ACTIVITY OF 1,4-NAPHTHOQUINONE DERIVATIVES AND QSAR STUDY

Veda Prachayasittikul[1, 2], Ratchanok Pingaew[3], Apilak Worachartcheewan[2,4,5], Somkid Sitthimonchai[6], Chanin Nantasenamat[2], Supaluk Prachayasittikul[2*], Somsak Ruchirawat[7,8], Virapong Prachayasittikul[1*]

[1] Department of Clinical Microbiology and Applied Technology, Faculty of Medical Technology, Mahidol University, Bangkok 10700, Thailand
[2] Center of Data Mining and Biomedical Informatics, Faculty of Medical Technology, Mahidol University, Bangkok 10700, Thailand
[3] Department of Chemistry, Faculty of Science, Srinakharinwirot University, Bangkok 10110, Thailand
[4] Department of Community Medical Technology, Faculty of Medical Technology, Mahidol University, Bangkok 10700, Thailand
[5] Department of Clinical Chemistry, Faculty of Medical Technology, Mahidol University, Bangkok 10700, Thailand
[6] Laboratory of Chemical Carcinogenesis, Chulabhorn Research Institute, Bangkok 10210, Thailand
[7] Laboratory of Medicinal Chemistry, Chulabhorn Research Institute and Program in Chemical Biology, Chulabhorn Graduate Institute, Bangkok 10210, Thailand
[8] Center of Excellence on Environmental Health and Toxicology, Commission on Higher Education (CHE), Ministry of Education, Thailand

* Corresponding authors: E-mail: supaluk@g.swu.ac.th, virapong.pra@mahidol.ac.th; Phone: +66 2 441 4371; Fax: +66 2 441 4380

http://dx.doi.org/10.17179/excli2017-309

This is an Open Access article distributed under the terms of the Creative Commons Attribution License (http://creativecommons.org/licenses/by/4.0/).

STRUCTURE-ACTIVITY RELATIONSHIPS OF THE COMPOUNDS 1–11: A DETAILED DISCUSSION

Structure-activity relationships (SAR) of the compounds **1–11** revealed that the chloro (Cl) group at C-2 position of compound **1** was replaced by amino group to give 2-substituted amino-3-chloro compounds (**2-11**) with lower to inactive aromatase inhibitory activities, except for compound **4** which still exhibited equipotent activity as compared to compound **1**. The 2-substituted amino groups were *N*-phenyl and *N*-alkyl of secondary amines, but compound **4** was tertiary amine. Moreover, the phenyl groups of 2-amino position constitute electron donating and electron withdrawing substituents (R) at *ortho*-, *meta*-, and *para*-positions. Obviously, the enhanced activity of inactive compound (**3**) was noted when R = electron donating (*N*-phenyl or anilinyl) to give compound **8** (IC_{50} = 1.9 µM) comparing to R = electron withdrawing groups ($COCH_3$ and COOH). It was found that *meta*-$COCH_3$ (**6**, IC_{50} = 3.1 µM) exhibited stronger activity than *para*-$COCH_3$ (**7**, IC_{50} = 5.3 µM) and *ortho*-COOH (**5**, IC_{50} = 18.0 µM).

In case of *N*-alkyl substituted compound (**2**, R = C_4H_9) and *N*-alkylaryl (**10**, R = CH_2CH_2Ph), the latter (**10**) displayed better activity than the former (**2**). When dimethoxy (di-OMe) groups were introduced to the phenyl (Ph) ring of compound **10,** compound **11** was obtained with totally loss of activity. It could be presumably suggested that $diOCH_3$ groups made the molecule (**11**) arrange in an inappropriate form in interacting with the target enzyme.

Interestingly, introducing methyl (CH_3) group to the secondary amine of inactive compound **3** resulted in the most potent tertiary amine compound (**4**). This could be possibly due to the lipophilicity and electron rich moiety of the amine **4**, which are required for the most potent activity comparing to the other secondary amines. Similarly, the most potent activity of compound **1** could be attributed to the electronic and lipophilic properties of the Cl group in enhancing the activity. In addition to *N*-phenyl moieties at position 2, *N*-quinolinyl compound **9** exhibited relatively high inhibitory activity (IC_{50} = 4.7 µM). Again, this could be possibly due to the hydrophobic effect of the condensed quinolinyl ring.

Table S1: Values of informative molecular descriptors of tested compounds (**1-11**) and virtually modified compounds (series **1-11**)

Compound	Mor04m	Mor08e	H8m	G1v	SIC2
**1**	1.226	-0.928	0.000	0.193	0.707
**2**	1.839	0.249	0.010	0.167	0.808
**3**	1.672	-0.494	0.001	0.169	0.673
**4**	1.104	-0.728	0.002	0.190	0.682
**5**	2.245	-0.182	0.053	0.165	0.755
**6**	1.885	-0.642	0.004	0.163	0.765
**7**	2.320	-0.280	0.005	0.179	0.751
**8**	1.499	-0.478	0.021	0.169	0.612
**9**	2.373	-1.001	0.039	0.179	0.770
**10**	1.799	-0.071	0.010	0.191	0.720
**11**	1.151	0.295	0.032	0.155	0.779
**1a**	0.479	-0.644	0.000	0.193	0.707
**1b**	-5.734	-0.583	0.000	0.193	0.707
**1c**	0.465	-0.757	0.000	0.193	0.707
**2a**	1.625	0.246	0.000	0.171	0.824
**2b**	1.664	-0.027	0.000	0.175	0.814
**2c**	1.322	0.839	0.013	0.163	0.802
**2d**	1.260	0.666	0.001	0.167	0.816
**2e**	1.315	0.560	0.000	0.171	0.805
**2f**	1.264	-0.132	0.000	0.175	0.726
**3a**	2.165	-1.458	0.024	0.177	0.675
**3b**	1.687	0.111	0.008	0.162	0.699
**4a**	0.692	-0.911	0.002	0.162	0.705
**4b**	0.845	-0.745	0.005	0.159	0.724
**4c**	1.743	-0.134	0.012	0.159	0.705

Table S1 (cont.): Values of informative molecular descriptors of tested compounds (**1-11**) and virtually modified compounds (series **1-11**)

Compound	Mor04m	Mor08e	H8m	G1v	SIC2
**4d**	0.753	0.100	0.019	0.169	0.723
**4e**	1.513	-1.588	0.030	0.163	0.683
**4f**	0.585	-1.436	0.071	0.151	0.700
**5a**	2.035	-0.693	0.003	0.165	0.775
**5b**	2.349	-0.444	0.002	0.165	0.760
**5c**	1.455	-1.125	0.088	0.162	0.773
**5d**	1.622	-0.686	0.005	0.162	0.760
**6a**	2.852	-0.960	0.018	0.163	0.765
**6b**	1.980	-1.536	0.106	0.160	0.764
**6c**	1.441	-0.938	0.039	0.174	0.764
**7a**	2.837	-1.022	0.003	0.193	0.751
**7b**	1.834	-1.161	0.007	0.160	0.752
**7c**	1.448	-0.472	0.007	0.174	0.752
**8a**	0.732	-0.473	0.012	0.186	0.644
**8b**	1.864	-0.558	0.002	0.156	0.627
**8c**	0.612	-0.835	0.037	0.165	0.659
**9a**	1.587	-0.726	0.025	0.163	0.781
**9b**	2.209	-0.575	0.024	0.179	0.763
**9c**	2.190	-0.814	0.032	0.179	0.752
**10a**	2.067	-0.591	0.031	0.165	0.699
**10b**	1.383	0.718	0.067	0.159	0.724
**10c**	1.361	-0.395	0.046	0.162	0.705
**11a**	0.575	0.629	0.091	0.153	0.778
**11b**	1.783	-0.222	0.035	0.166	0.776
**11c**	0.665	0.326	0.111	0.173	0.775

Table S2: Experimental and predicted aromatase inhibitory activities (pIC_{50}) of compounds **1-11**

Compound	Experimental activity	Predicted activity	Residual
**1**	0.301	0.348	0.047
**2**	-0.940	-1.013	-0.073
**3**	- [a]	- [a]	- [a]
**4**	0.301	0.249	-0.052
**5**	-1.255	-1.205	0.050
**6**	-0.491	-0.420	0.071
**7**	-0.724	-0.763	-0.039
**8**	-0.279	-0.324	-0.045
**9**	-0.672	-0.737	-0.065
**10**	-0.519	-0.467	0.052
**11**	- [a]	- [a]	- [a]

[a]The compound was experimentally inactive and was excluded from the data set of QSAR analysis.

EXCLI Journal 2017;14:714-726 – ISSN 1611-2156
Received: March 28, 2017, accepted: May 02, 2017, published: May 16, 2017

Table S3: Effects of substituents on 2-amino-3-chloro-1,4-naphthoquinone core structure on important descriptor values and aromatase inhibitory activity

Panel	Substituent at position 2 (compound)	Modification effect[a]	Altered descriptors
**1**	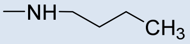 (**2**)	↓ activity: **2** < **1**	↑: Mor04m, Mor08e<br>↓: G1v
**2**	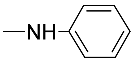 (**3**)	↓ activity: **3** (IA) < **1** (IA)	↑: Mor04m, Mor08e<br>↓: G1v
**3**	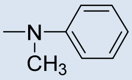 (**4**)	$-CH_3$: ↑ activity : **4** > **3**	↑: G1v<br>↓: Mor04m, Mor08e
**4**	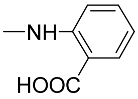 (**5**)	-COOH : ↓ activity : **5** < **4**	↑: Mor04m, Mor08e, H8m<br>↓: G1v
**5**	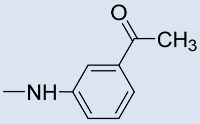 (**6**)	*m*-$COCH_3$: ↓ activity : **6** < **4**	↑: Mor04m, Mor08e, H8m<br>↓: G1v
**6**	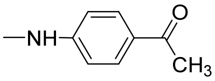 (**7**)	*p*-$COCH_3$: ↓ activity: **7 < 4**	↑: Mor04m, Mor08e, H8m<br>↓: G1v
**7**	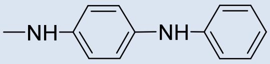 (**8**)	$-C_6H_6N$: ↓ activity : **8** < **4**	↑: Mor04m, Mor08e, H8m<br>↓: G1v
**8**	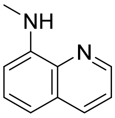 (**9**)	Quinolinyl : ↑ activity :<br>**9** > **2**, **9** > **3**	↑: Mor04m, H8m, G1v<br>↓: Mor08e
**9**	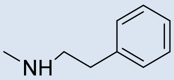 (**10**)	Phenyl ring: ↑ activity : **10** > **2**	↑: G1v<br>↓: Mor04m, Mor08e
**10**	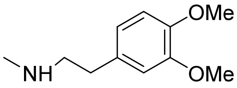 (**11**)	-OMe : ↓ activity :<br>**11** (IA) < **10** (A)	↑: Mor08e<br>↓: Mor04m, G1v

IA = inactive, A = active. [a] Experimental activity of the tested compounds was used for comparison. The deteriorate effects were found in all tested compounds when compared to the prototype **1**, except for compound **4**.

Table S4: Predicted aromatase inhibitory activity (pIC_{50}) of virtually modified compounds (series **1-11**) and experimental aromatase inhibitory activity of reference drugs

Compound	pIC_{50}	Compound	pIC_{50}
**1a**	0.550[a]	**6a**	-0.898[b]
**1b**	3.582[a]	**6b**	-0.898[b]
**1c**	0.614[a]	**6c**	-0.294[b]
**2a**	-0.758[b]	**7a**	-0.494[b]
**2b**	-0.599[b]	**7b**	-0.217[b]
**2c**	-1.047[c]	**7c**	-0.265[b]
**2d**	-0.819[b]	**8a**	0.259[a]
**2e**	-0.742[b]	**8b**	-0.390[b]
**2f**	-0.253[b]	**8c**	0.124[a]
**3a**	-0.151[b]	**9a**	-0.465[b]
**3b**	-0.718[b]	**9b**	-0.696[b]
**4a**	0.326[a]	**9c**	-0.618[b]
**4b**	0.099[a]	**10a**	-0.712[b]
**4c**	-0.684[b]	**10b**	-1.391[c]
**4d**	-0.311[b]	**10c**	-0.611[b]
**4e**	0.071[a]	**11a**	-1.243[c]
**4f**	0.016[a]	**11b**	-0.865[b]
**5a**	-0.506[b]	**11c**	-1.136[c]
**5b**	-0.762[b]	Ketoconazole	-0.415[d]
**5c**	-0.698[b]	Letrozole	3.482[d]
**5d**	-0.329[b]		

[a] Highly active compound
[b] Moderately active compound
[c] Weakly active to inactive compound
[d] Experimental activity

Received: March 28, 2017, accepted: May 02, 2017, published: May 16, 2017

Table S5: Summary of structurally modified compounds (series **1-11**)

Series	Comparison	Affected de-scriptors	Related properties	Notes
**1**	Disubstitution at C-2 and C-3 position: ↑activity<br>di-I > di-F > di-Br> di-Cl<br>(**1b** > **1c** > **1a** > **1**)	Mor04m[a]<br>Mor08e	Mass<br>Electronegativity	**1b**[b] is the most potent of the series 1.<br>All modifications ↑activity.<br>Disubstitution with high EN atoms can markedly improve activity. All compounds in series **1** had the same values of H8m = 0, G1v = 0.193 and SIC2 = 0.707
**2**	Length of substituted alkyl chain on amino group :<br>short chain > long chain<br>(**2f** > **2b** > **2e** > **2a** > **2d** > **2** > **2c**)	Mor08e[a]<br>Mor04m<br>G1v	Electronegativity<br>Mass<br>*van der* Waals volume	**2f**[b] is the most potent of the series 2.<br>All modifications ↑activity, except **2c**.<br>Dimethyl substitution (**2f**) provide the best activity than long alkyl chain.
	CH_3 substitution on NH group: ↓activity<br>(**2b** > **2e** , **2a** > **2d**, **2** > **2c**)	Mor08e[c]	Electronegativity	Within the same length of alkyl chain, the ones with additional CH_3 substitution on amino group are less potent.
**3**	Type of substituted ring on NH group :<br>1-adamatanyl > cyclo-hexyl > phenyl<br>(**3a** > **3b** > **3**)	Mor04m<br>Mor08e[c]<br>G1v	Mass<br>Electronegativity<br>*van der* Waals volume	**3a**[b] is the most potent of the series 3.<br>All modifications ↑activity.<br>Substitution with 1-adamantyl group pro-vides the best activity.
**4**	Within the same length of substituted alkyl chain on N atom:<br>phenyl > 1-adamantyl > cyclohexyl<br>(**4a** > **4f** > **4d**)	Mor04m<br>Mor08e[c]<br>G1v	Mass<br>Electronegativity<br>*van der* Waals volume	**4a**[b] is the most potent of the series 4.<br>All modifications ↓activity, except **4a**.<br>Phenyl derivative substituted with 2C alkyl chain provided the most potent activity.
	Length of substituted alkyl chain for phenyl and cyclohexyl :<br>2C > 1C<br>(**4a** > **4** & **4d** > **4c**)	Mor04m<br>Mor08e[c]	Mass<br>Electronegativity	Appropriate chain length in combination with distinct type of substituted ring are es-sential for potent activity.
	Length of substituted alkyl chain for 1-ada-mantyl: 1C > 2C<br>(**4e** > **4f**)	Mor08e<br>G1v<br><br>H8m	Electronegativity<br>*van der* Waals volume<br>Mass	Appropriate chain length in combination with distinct type of substituted ring are es-sential for potent activity.
**5**	Position of COOH on phenyl ring of 2-amino group:<br>*meta* > *para* > *ortho*<br>(**5a** > **5b** > **5**)	H8m<br>Mor04m<br>Mor08e	Mass<br>Mass<br>Electronegativity	**5d**[b] is the most potent of the series 5.<br>All modifications ↑activity.<br>Equal G1v values were observed among **5**, **5a** and **5b**.<br>Particular position of COOH substitution on the phenyl ring together with the presence of *N*-CH_3 moiety influence the activity (**5d** > **5a** > **5c** > **5b** > **5**).
	CH_3 substitution on NH group:<br>*meta* series:↓activity<br>(**5a** > **5c**)<br>*para* series: ↑activity<br>(**5d** > **5b**)	H8m<br>Mor04m<br>Mor08e	Mass<br>Mass<br>Electronegativity	Introducing CH_3 group to *para*-COOH com-pound improves activity whereas reduced activity is observed in *meta*-COOH com-pound.<br>Equal G1v values were observed among **5c** and **5d**.

[a] Shift from positive to negative value was observed. [b] The most potent of all tested and modified com-pounds. [c] Shift from negative to positive value was observed.

Table S5 (cont.): Summary of structurally modified compounds (series **1-11**)

Series	Comparison	Affected de-scriptors	Related properties	Notes
**6**	CH_3 substitution on NH group of *m*-ketone:<br>NCH_3 series: ↑activity (**6c** > **6**)<br>NH and NCH_3 series of $COCF_3$ ketone: no effect (**6a** = **6b**)	Mor04m<br>Mor08e<br>H8m<br>G1v	Mass<br>Electronegativity<br>Mass<br>*van der* Waals volume	**6c**[b] is the most potent of the series 6.<br>All modifications ↓activity, except **6c**.<br>The substitution of CH_3 on amino group of *m*-ketone improves activity.
	Type of substituted *m*-ketone:<br>$COCH_3$ > $COCF_3$<br>(**6** > **6a**)	Mor04m<br>Mor08e<br>H8m	Mass<br>Electronegativity<br>Mass	Substitution with high electronegativity (EN) moiety affects mass (↑Mor04m and ↑H8m) and electronegativity (↓Mor08e) descriptors leading to reduced activity.<br>Equal values of G1v and SIC2 descriptors were observed for **6** and **6a** indicating that *van der* Waals (G1v) and structural information (SIC2) descriptors are not altered by substitution with high EN moiety.
**7**	CH_3 substitution on NH group of *p*-ketone:<br>NCH_3 of p-$COCF_3$ series: ↑activity (**7b** > **7a**)<br>NCH_3 of p-$COCH_3$ series:↑activity (**7c > 7**)	Mor04m<br>Mor08e	Mass<br>Electronegativity	**7b**[b] is the most potent of the series 7.<br>All modifications ↑activity.<br>*N*-CH_3 substitution of *p*-$COCF_3$ and *p*-$COCH_3$ improves activity. Marked improved activity was observed in case of **7c**.
	Type of substituted *p*-ketone:<br>$COCF_3$ > $COCH_3$<br>(**7a** > **7**)	Mor04m<br>G1v<br><br>Mor08e<br>H8m	Mass<br>*van der* Waals volume<br>Electronegativity<br>Mass	Substitution with high EN moiety affects mass (↑Mor04m), electronegativity (↓Mor08e) and *van der* Waals volume (↑G1v) descriptors and leads to improved activity.<br>The effect found in series **7** is in contrast to series **6**.
**8**	Position of anilinyl group on phenyl ring: *para* > *meta*<br>(**8** > **8b**)	Mor04m<br>G1v<br><br>SIC2	Mass<br>*van der* Waals volume<br>Structural information	**8a**[b] is the most potent of the series 8.<br>All modifications ↑activity, except **8b**.<br>*Para*-substitution of anilinyl (C_6H_6N) provides better activity than *meta*-substitution.
	CH_3 substitution on NH group:<br>*para*- and *meta*- series: ↑activity<br>(**8a** >**8** and **8c** > **8b**)	Mor04m<br>G1v	Mass<br>*van der* Waals volume	Introducing CH_3 group to the aminophenyl moiety (both *para*- and *meta-)* derivatives can markedly improve activity (shift to positive pIC_{50} values).
**9**	Position of substituted aminoquinolone (AQ) ring:<br>5AQ > 2AQ > 8AQ > 4AQ<br>(**9a** > **9c** > **9** > **9b**)	Mor04m<br>Mor08e	Mass<br>Electronegativity	**9a**[b] is the most potent of the series 9.<br>All modifications ↑activity, except **9b**.<br>Marked decrease of Mor08e and Mor04m values were observed in the most potent compound of the series (the 5AQ derivative, **9a**).<br>The substituted position of AQ ring affects mass and electronegativity descriptors.

[a] Shift from positive to negative value was observed. [b] The most potent of all tested and modified compounds. [c] Shift from negative to positive value was observed.

EXCLI Journal 2017;14:714-726 – ISSN 1611-2156
Received: March 28, 2017, accepted: May 02, 2017, published: May 16, 2017

Table S5 (cont.): Summary of structurally modified compounds (series **1-11**)

Series	Comparison	Affected de-scriptors	Related properties	Notes
**10**	Length of alkyl chain linked between amino group and phenyl ring: 2C > 1C (**10** > **10a**)	Mor04m<br>H8m<br>G1v	Mass<br>Mass<br>*van der* Waals volume	All modifications ↓activity.<br>The most potent compound of the series is the prototype **10**[b].<br>2C chain length is noted as the most appropriate linker (**10**).
	CH_3 substitution on NH group:<br>1C length:↑activity (**10c** > **10a**)<br>2C length:↓activity (**10** > **10b**)	Mor04m<br>Mor08e[c]<br>H8m	Mass<br>Electronegativity<br>Mass	Substitution of CH_3 moiety on the 2-amino group can improve or reduce activity of the compound depending on the length of linker chain.
**11**	Position of diOMe on the phenyl ring : 2,3-position > 3,4-position (**11b** > **11**)	Mor08e[a]<br>G1v	Electronegativity<br>*van der* Waals volume	**11b** [b] is the most potent of the series 11.<br>All modifications ↑activity.<br>Distinct position of diOMe substitution on the phenyl ring is required for preferable activity.
	CH_3 substitution on NH group:<br>2,3-diOMe: ↓activity (**11b** > **11c**)<br>3,4-diOMe: ↑activity (**11a** > **11**)	Mor08e[c]<br>Mor04m<br>H8m	Electronegativity<br>Mass<br>Mass	Substitution of CH_3 moiety on the 2-amino group can improve or reduce activity of the compound depending on the position of di-OMe substitution.

[a] Shift from positive to negative value was observed. [b] The most potent of all tested and modified compounds. [c] Shift from negative to positive value was observed.

EXCLI Journal 2017;14:714-726 – ISSN 1611-2156
Received: March 28, 2017, accepted: May 02, 2017, published: May 16, 2017

PREDICTION OF STRUCTURALLY MODIFIED COMPOUNDS USING CONSTRUCTED QSAR MODEL: A DETAILED DISCUSSION

The effect of substituted alkyl chain length on the amino group was observed in modified compounds series **2** in which all of them exhibited higher activity than the parent compound **2**, except for compound **2c**. It was found that the shorter substituted alkyl chain provided the better activity than the long chain alkyl group *via* an increased *van der* Waals volume (G1v) but mostly with decreased mass (Mor04m) and electronegativity (Mor08e) as seen in **2b** > **2a** > **2**, Table S1. Particularly, *N,N*-dimethylaminoquinone **2f** was found to be the most potent compound (Table S4). This could be due to high G1v value of 0.175 that gave 1st component symmetry directional WHIM index deriving from dimethyl substituents on the amino moiety. Furthermore, the additional CH_3 substituent at amino group gave tertiary amines with reduced activity when compared to compounds with the same length of substituted alkyl chain (**2b** > **2e**, **2a** > **2d** and **2** > **2c**, Table S4).

The notable enhanced effects of ring substituted at the amino group was observed in modified compounds series **3** in which the replacement of phenyl ring of **3** by 1-adamantyl and cyclohexyl rings can markedly increase the activity of the inactive compound (**3**) to moderately actives **3a** and **3b**, respectively. Furthermore, the substitution of various quinolines at 2-amino position in modified series **9** provided more potent compounds as compared to the *N*-alkyl chain and *N*-phenyl substitutions in series **2** and **3**, respectively. Compound **9a** was predicted to be the most potent compound of the series **9**, which indicated the substitution at position 5 of quinoline ring to be the most appropriate substitution. It was noticed that all aminoquinoline derivatives in series **9**, except for **9a**, possess equivalent G1v values of 0.179 (Table S1). The exceptional activity of 5-aminoquinoline compound **9a** may be due to its decreased mass (Mor04m), electronegativity (Mor08e) and *van der* Waals volume (G1v) but increased neighborhood symmetry of 2-order (SIC2).

The effects of *ortho-*/*meta-*/*para-*carboxyl (COOH) and acetyl ($COCH_3$) substituted on amino phenyl ring along with the presence of additional methyl (CH_3) substituent on 2-amino group of 1,4-naphthoquinone core were investigated as shown in modified compounds series **5**, **6**, and **7**. Interestingly, the addition of CH_3 moiety can either improve or reduce the activity of the compound depending on the position of substituted carboxyl (COOH) and acetyl ($COCH_3$) groups, Table S4. For carboxyl compounds (series **5**), compound **5d** exhibited the best activity (moderately active, pIC_{50} -0.329) affording 1.26 folds more potent than the reference drug, ketoconazole (pIC_{50} -0.415) which indicated the *para*-carboxyl substitution on the phenyl ring together with the insertion of methyl moiety to 2-amino group as the most appropriate modification of the series. For acetyl compounds (series **6** and **7**), the *meta*-substituted compound (**6**) exhibited more potent activity than that of the *para*-substituted compound (**7**). In addition, introducing the methyl moiety to the 2-amino group of the compound can markedly improve activity of both *meta-* and *para*-compounds (**6c** > **6**: 1.67 folds and **7c** > **7**: 2.73 folds, respectively). In addition, the effect of replacing the acetyl ($COCH_3$) moiety with the trifluoroacetyl ($COCF_3$) moiety were investigated. The contrast effects were observed in *meta-* (**6**) and *para-* (**7**) series. Introducing the $COCF_3$ moiety to the compound increased activity of the *para*-compound (**7a** > **7**: 1.47 folds) whereas vice versa effect was observed in case of the *meta*-compound (**6** > **6a**: 1.83 folds). Moreover, no change was observed when methyl group was added to 2-amino group of *meta*-trifluoroacetyl compound **6a** (**6a** = **6b**) but improved activity was obtained in case of the *para*-trifluoroacetyl compound **7a** (**7b > 7a**: 2.28 folds).

It was found that the length of alkyl chain linking between 2-amino group and the terminal phenyl ring influences the activity of the compounds. Considering the structural modifications of compound series **10**, the improved activity cannot be found in the modified compounds (i.e., **10a**, **10b** and **10c**), however, the parent compound **10** with 2C chain length linker was observed

to be the most appropriate chain length providing the best activity. Moreover, the effect of additional methyl substituent at 2-amino group on activity of compounds was dependent on the length of the linker chain (Table S4). The effects of diOMe substituents on the terminal phenyl ring were observed along with the presence of additional methyl substitution at 2-amino group (series **11**). All modified compounds in this series were predicted to exhibit improved activity (moderately to weakly active) as compared to their parent compound **11** (inactive). It was found that more potent activity can be achieved by 2,3-diOMe substitution (**11b**) rather than by 3,4-diOMe substitution (**11**). The enhanced effect may be governed by the decreasing of electronegativity descriptor value (Mor08e: **11 =** 0.295, **11b** = -0.222) in which the shift from positive to negative value was observed for 2,3-diOMe substituted compound (**11b**). Substitution by methyl moiety on the 2-amino group of 3,4-diOMe compound (**11**) gave compound **11a** with the improved activity *via* marked decrease of Mor04m (2.00 folds), and marked increase of Mor08e (2.13 folds) and H8m (2.84 folds) descriptor values as compared to the parent compound **11**. In contrast, the deteriorate effect of methyl substitution on the 2-amino group was observed in case of the most potent 2,3-diOMe compound (**11b**) was converted to *N*-methyl compound **11c**. In this regard, a pronounced increase of Mor08e value of **11c** was observed (Mor08e: **11b** = -0.222 and **11c** = 0.326) along with an increase of H8m (3.17 folds) and a decrease of Mor04m (2.68 folds).
